# Vitamin D Biofortification of Pork May Offer a Food-Based Strategy to Increase Vitamin D Intakes in the UK Population

**DOI:** 10.3389/fnut.2021.777364

**Published:** 2021-12-03

**Authors:** Holly R. Neill, Chris I. R. Gill, Emma J. McDonald, W. Colin McRoberts, L. Kirsty Pourshahidi

**Affiliations:** ^1^Nutrition Innovation Centre for Food and Health, School of Biomedical Sciences, Ulster University, Coleraine, United Kingdom; ^2^Devenish Nutrition Ltd., Belfast, United Kingdom; ^3^Agri-Food and Biosciences Institute, Belfast, United Kingdom

**Keywords:** cholecalciferol, 25-hydroxyvitamin D (25(OH)D), National Diet and Nutrition Survey, dietary modeling, bio-addition, meat, feed supplementation, UVB radiation

## Abstract

Hypovitaminosis D is prevalent worldwide, with many populations failing to achieve the recommended nutrient intake (RNI) for vitamin D (10–20 μg/day). Owing to low vitamin D intakes, limited exposure to ultraviolet-B (UVB) induced dermal synthesis, lack of mandatory fortification and poor uptake in supplement advice, additional food-based strategies are warranted to enable the UK population to achieve optimal vitamin D intakes, thus reducing musculoskeletal risks or suboptimal immune functioning. The aims of the current study were to (1) determine any changes to vitamin D intake and status over a 9-year period, and (2) apply dietary modeling to predict the impact of vitamin D biofortification of pork and pork products on population intakes. Data from the UK National Diet and Nutrition Survey (Year 1–9; 2008/09–2016/17) were analyzed to explore nationally representative mean vitamin D intakes and 25-hydroxyvitamin D (25(OH)D) concentrations (*n* = 13,350). Four theoretical dietary scenarios of vitamin D pork biofortification were computed (vitamin D content +50/100/150/200% vs. standard). Vitamin D intake in the UK population has not changed significantly from 2008 to 2017 and in 2016/17, across all age groups, 13.2% were considered deficient [25(OH)D <25 nmol/L]. Theoretically, increasing vitamin D concentrations in biofortified pork by 50, 100, 150, and 200%, would increase vitamin population D intake by 4.9, 10.1, 15.0, and 19.8% respectively. When specifically considering the impact on gender and age, based on the last scenario, a greater relative change was observed in males (22.6%) vs. females (17.8%). The greatest relative change was observed amongst 11–18 year olds (25.2%). Vitamin D intakes have remained stable in the UK for almost a decade, confirming that strategies are urgently required to help the population achieve the RNI for vitamin D. Biofortification of pork meat provides a proof of concept, demonstrating that animal-based strategies may offer an important contribution to help to improve the vitamin D intakes of the UK population, particularly adolescents.

## Introduction

Substantial evidence exists to suggest hypovitaminosis D is prevalent globally and, assuming minimal sunlight exposure, many also fail to achieve the recommended nutrient intake (RNI) for vitamin D (10–20 μg/day) (1–3). Within the UK, one in five adults do not meet the RNI of 10 μg/day which is currently advised by the Scientific Advisory Committee on Nutrition (4) to reduce the risk of musculoskeletal diseases (5, 6). Therefore, a large proportion of the population have suboptimal 25-hydroxyvitamin D (25(OH)D) concentrations (a reliable and robust blood marker of vitamin D status). The exact 25(OH)D concentration classified as deficient is disputed and ranges from <25 to <50 nmol/L (4, 5, 7). The reason for suboptimal vitamin D status is multifactorial, owed predominantly to limited natural food sources, lack of mandatory fortification, poor implementation of supplement advice, especially amongst low socioeconomic groups, and insufficient dermal synthesis from ultraviolet-B (UVB) exposure (8, 9). Thus, additional sustainable food-based strategies are urgently warranted to enable populations to achieve adequate vitamin D intakes (10).

Fortification and biofortification (also referred to as “bio-addition”) are popular initiatives to help alleviate vitamin deficiencies globally; the former being widely accepted by consumers nowadays and a plethora of research confirming its effectiveness (11–13). Vitamin D fortification in particular is well-established as a method to improve consumer vitamin D intakes and subsequently increase circulating 25(OH)D concentrations (14–16). Many have argued the benefits and feasibility of vitamin D fortification policies, considering both the efficacy and safety of various vehicles and scenarios (16–20). Countries having implemented mass vitamin D fortification policies observed increased 25(OH)D concentrations and reduced rates of deficiency in their populations (21–23). However, within the UK, vitamin D fortification is applied on a voluntary basis, with the exception of infant formula. On a population level, mandatory biofortification and fortification presents as the only method to increase 25(OH)D concentrations within the general population.

Biofortification refers to the endogenous increase of nutrients during the growth phase, either in animal-based or plant-based foods. Evidence has shown the plausibility of increasing the vitamin D content in commodity animal-based foods such as pork, beef, chicken and eggs, as well as mushrooms (24). Amongst animal foods, vitamin D biofortification can be achieved by either UVB radiation and/ or feed alteration. Whilst oily fish such as salmon, herring, sardines and mackerel are one of the richest sources of vitamin D_3_ (~3–19 μg/100 g) (25), in general, its consumption remains unpopular in the UK owing to a variety of factors including taste preferences, perceived cost and sustainability concerns (26). Currently, meat and meat products remain the main contributor (30%) to vitamin D intakes in the UK adult (19–64 years) diet (27), despite relatively lower vitamin D contents per 100 g (~0.1–1.9 μg/100 g) (25). In particular, pork is popular amongst the UK population and is the most widely consumed meat worldwide (28). Typically, pig feed in the UK contains the maximum permitted concentration of 50 μg (2,000 IU) vitamin D/kg owing to animal health benefits; albeit this is considered substandard by many, and revaluation has been proposed (29). Thus, owing to dietary supplementation restrictions, UVB exposure is warranted to further elevate vitamin D concentrations in pork meat. As such, enriching common foods, especially meat and meat products, may complement the diet of at-risk populations.

Dietary mathematical modeling can predict the impact of vitamin D biofortification implementation and has been previously conducted to assess differing vitamin D fortification scenarios (17–19, 30, 31). However, to the authors knowledge, this is the first study to investigate the theoretical impact of biofortified meat.

Therefore, the aims of the current study were (1) quantify vitamin D intake and status over a 9-year period and; (2) use dietary modeling scenarios to predict how biofortification of pork meat could improve vitamin D intakes across the UK population.

## Materials and Methods

### Study Population

Participants from the UK National Diet and Nutrition Survey Rolling Program (NDNS) Years 1–9 (2008/09–2016/17) dataset were used in the present manuscript. Details regarding the design, participant selection, recruitment process and data collection of NDNS are reported in full elsewhere (32–36). In short, jointly funded by Public Health England and the Food Standards Agency, NDNS is a UK-wide continuous cross-sectional survey. Fieldwork began in 2008 and provides quantitative comprehensive information regarding diet, nutritional status, sociodemographic characteristics, lifestyle, and physical activity levels from a nationally representative sample of the general UK population aged 1.5 years and older. Using household post-code details from the Post-code Address File, participants were stratified and randomly recruited to take part. All food and drink consumption were estimated by participants using a self-reported 3 or 4-day food diary. Nutrient intakes were then quantified using McCance and Widdowson's The Composition of Foods Integrated Dataset (CoFID). Following written consent, fasting blood samples were collected by venepuncture and transported in a cool box to a local processing field laboratory within 2 h of blood collection. All samples were centrifuged at 2,000 g for 20 min at 4°C and aliquoted. Survey years 1–6 (2008–2014) used competitive chemiluminescence immunoassay (CLIA) to determine plasma and serum 25(OH)D, whilst Years 7–9 (2014–2017) measured serum 25(OH)D by liquid chromatography coupled to tandem mass spectrometry (LC-MS/MS). Quality controls were included within each batch of samples and National Institute of Standards and Technology (NIST) confirmed accuracy. Involvement in blood sampling was lower than other aspects of NDNS observations and therefore, participant numbers are inconsistent between vitamin D intake and status. Ethical approval was obtained from Oxfordshire Research Ethics Committee (Ref. No. 07/H0604/113) and data were made available from the UK Data Archives (37).

### Data Modeling

As meat and meat products are one of the main contributors (18–31%) to vitamin D intakes within the UK population (27), pork and pork products were selected for predictive dietary modeling (see full list of food codes in Supplementary Material). The theoretical percentage increases from biofortification were selected based upon prior on-farm UVB exposure biofortification work in pigs (38, 39). Owing to varying concentrations of vitamin D in pork and pork products, biofortification increases were considered on a percentage basis. As such, in addition to current standard vitamin D content (baseline, 0%), four scenarios were examined to determine the effect of pork biofortification: 50, 100, 150, and 200% increase in total vitamin D concentrations in pork. Within the NDNS dataset, vitamin D is defined as total vitamin D, including both forms of vitamin D_3_ and D_2_. Composite dishes which did not include a significant proportion of pork were not included. Scenarios including and excluding supplements were both explored, as well as subgroup analyses of sex and age groups (1.5–3, 4–10, 11–18, 19–64, 65+ years).

### Data Analysis

Analysis of all data were performed using the Statistical Package for the Social Science for Windows (IBM SPSS Statistics version 25, Chicago IL, USA). All values are expressed as mean ± standard deviation (SD), unless otherwise specified. Descriptive statistics were used to present participant characteristics, mean vitamin D intakes and 25(OH)D concentrations. Normality tests were conducted for all data using Kolmogorov-Smirnov testing and, where necessary, data was log transformed. Independent *t*-tests and one-way analysis of variance (ANOVA) with *post-hoc* Tukey test were performed to calculate subgroup analysis in sex, age and season for vitamin D intakes and 25(OH)D concentrations. Current baseline total vitamin D intakes were calculated per participant and then expressed as a daily average based on the number of completed food diary days. The relevant pork food codes were identified from NDNS datasets (Supplementary Material) and a SPSS syntax was created to calculate the new vitamin D content of these foods based on our four scenarios (+50, 100, 150, and 200%). These values were applied to individual food intake data to create four new total vitamin D intakes for each participant. This data was again divided by the number of completed food diary days. One-way within-subjects repeated measures ANOVA and *post-hoc* pairwise comparisons were conducted to identify significance differences in daily vitamin D intakes between the various dietary modeling scenarios. Relative percentage change was calculated as below.


$$\begin{array}{l} Relative\;percentage\;change\\ \;=\frac{\begin{array}{c} vitamin\;D\;intake\;from\;biofortified\;pork\;-\\ vitamin\;D\;intake\;from\;standard\;pork \end{array}}{vitamin\;D\;intake\;from\;standard\;pork}\;x\;100 \end{array}$$

Values of *p* < 0.05 were deemed statistically significant throughout and results displayed in tabular and graphical form.

## Results

The study included 13,350 participants (46% males, 54% females), ranging in age from 1.5 to 96 years and residing in England (57.6%), Northern Ireland (13.6%), Scotland (15.5%), and Wales (13.3%). Participant characteristics are summarized in Table 1. The majority of participants never smoked (56.0%) while 21.9 and 21.2% either currently smoked or were ex-smokers, respectively.

**Table 1:** Participant characteristics from the UK National Diet and Nutrition Survey (NDNS) Years 1–9 (2008–2017).

	**All**	**Male**	**Female**	*P*-value^*^
	**(*n* = 13,350)**	**(*n* = 6,161)**	**(*n* = 7,189)**	
Age (y)	30 ± 24	28 ± 24	32 ± 24	<0.001
Weight (kg)	58.5 ± 27.3	59.9 ± 30.1	57.3 ± 24.7	NS
Height (m)	1.53 ± 0.24	1.56 ± 0.27	1.51 ± 0.22	<0.001
BMI (kg/m^2^)	23.5 ± 6.5	23.0 ± 6.2	24.0 ± 6.7	<0.001
Supplement user (%)	21.6	19.0	23.9	<0.001

*Data is presented as mean ± standard deviation, unless otherwise specified*.

* *P-value difference within rows between male and female participants; independent samples t-test on log transformed data, where required. Significance set at p < 0.05 throughout. UK, United Kingdom; n, number of participants; y, years; kg, kilograms; m, meters; BMI, body mass index; NS, not significant*.

### Vitamin D Intakes

**Figure 1:**
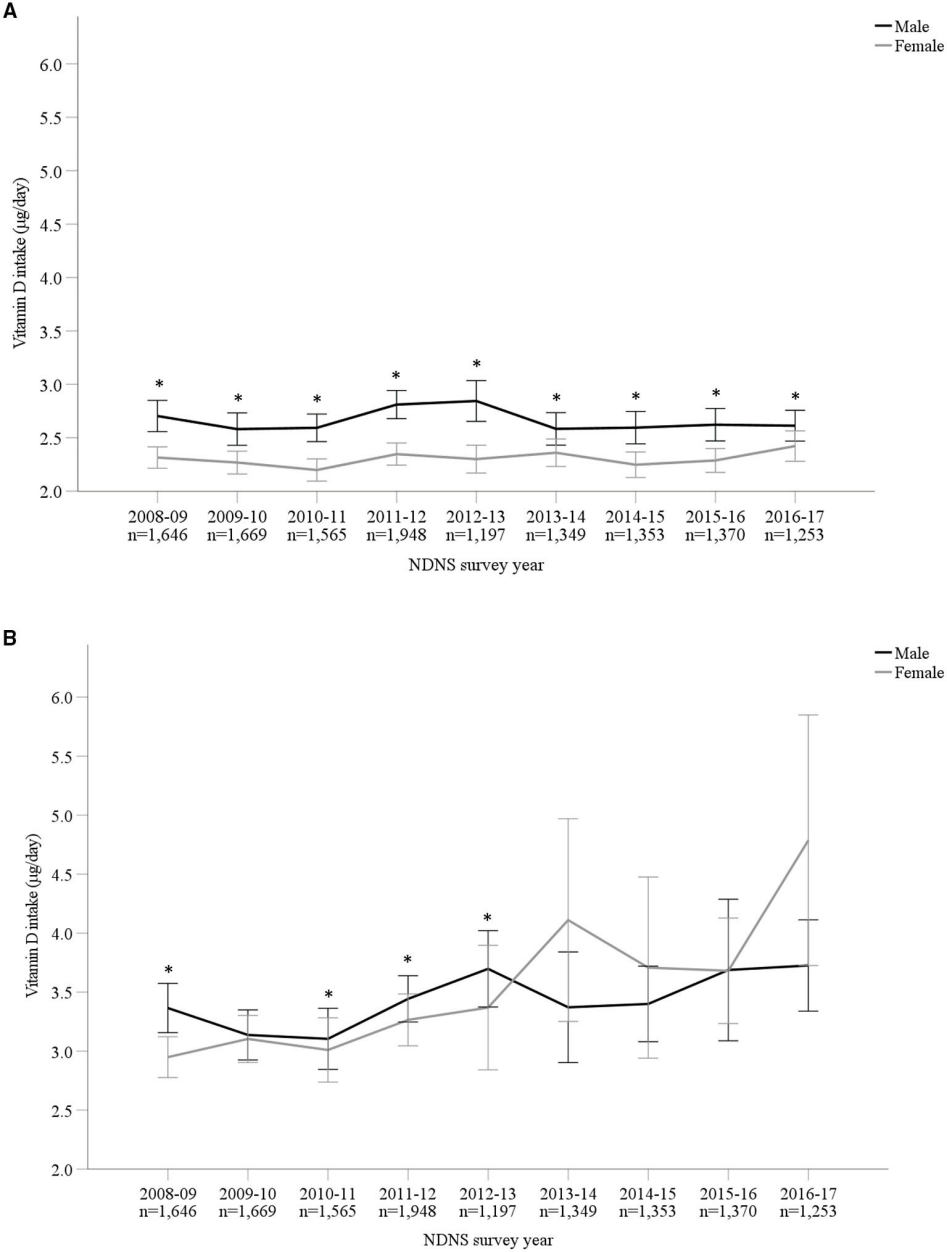
**(A,B)** Vitamin D intake (μg/day) from diet alone **(A)** and in combination with supplements **(B)** from Years 1–9 (2008–2017) of the UK National Diet and Nutrition Survey (NDNS). Data is presented as mean (95% CI). *Denotes significant difference (*p* < 0.05) between male and female participants; independent samples *t*-test using log transformed data. No significant difference (*p* > 0.05) between survey years in total group or within each gender; one-way ANOVA tests using log transformed data. UK, United Kingdom; *n*, number of participants; y, years; CI, confidence interval.

**Figure 2:**
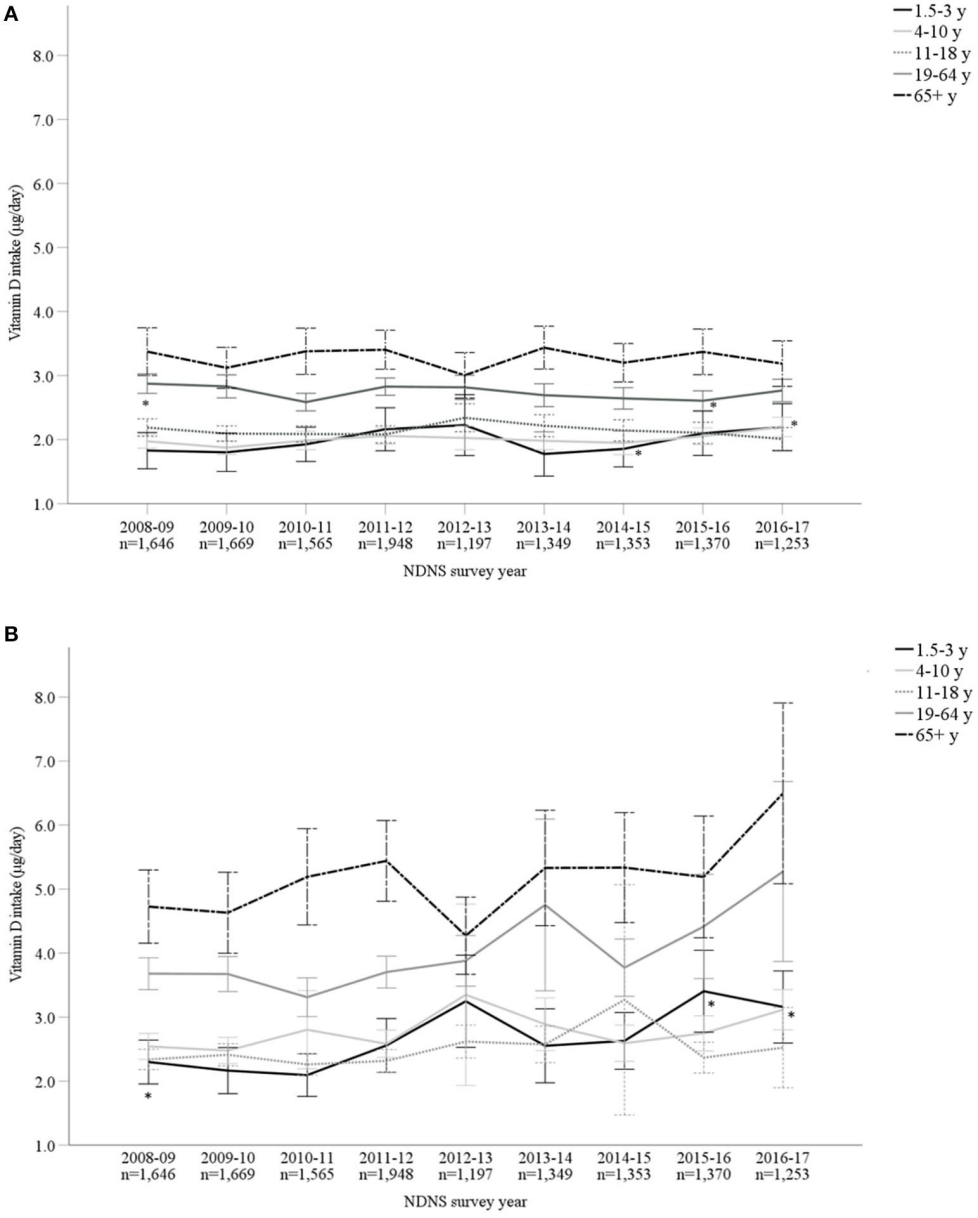
**(A,B)** Vitamin D intake (μg/day) from diet alone **(A)** and in combination with supplements **(B)**, split by age categories, from Years 1–9 (2008–2017) of the UK National Diet and Nutrition Survey (NDNS; total *n* = 13,350). Data is presented as mean (95% CI). *Denotes significant difference (*p* < 0.05) between survey years within age group; one-way ANOVA tests using log transformed data. For *post-hoc* (Tukey) tests between ages groups in the same survey year, see Supplementary Material. UK, United Kingdom; *n*, number of participants; y, years; CI, confidence interval.

Vitamin D mean intakes have not changed significantly between 2008 to 2017 in the UK population when considering diet alone or in combination with supplement intake (*p* > 0.05; Figures 1A,B; Supplementary Material). Including supplemental intake, 95.8% of participants failed to achieve the recommendation of 10 μg/day. The mean vitamin D intake for those below the RNI was 2.76 ± 1.99 μg/day. Participants consuming 10 μg or above (4.2%) daily vitamin D had mean intakes of 19.35 ± 24.29 μg/day. When considering diet alone, males reported a significantly higher mean vitamin D intake compared to females over all 9 years combined (M 2.66 ± 1.99 μg/day and F 2.30 ± 1.66 μg/day, *p* < 0.05) as well as each individual survey year. However, females reported greater mean daily vitamin D intakes in comparison with males when both diet and supplements were included overall years combined (M 3.42 ± 4.42 μg/day and F 3.50 ± 7.59 μg/day, *p* < 0.05). Figures 2A,B and Supplementary Material outline intakes from age groups in each survey year. In general, those aged 65 years and over consumed the highest amounts of vitamin D, whilst the lowest was most commonly observed in those aged 1.5–3 years. Some significant differences were observed in 4–10 years (2014/15 vs. 2016/17) and 19–64 years (2008/09 vs. 2015/16) from diet alone, and 1.5–3 years (2009/10 vs. 2015/16 and 2016/17) from diet and supplementation.

### Dietary Modeling Scenarios

Across all participants, our modeling scenarios demonstrated that a 5, 10, 15, or 20% increase in population vitamin D intake was achievable if the concentrations in biofortified pork were elevated by 50, 100, 150, and 200%, respectively (Table 2). Considering the 200% increase scenario, a greater relative change was observed in males (22.6%) compared to females (17.8%) (Table 2), and although older adults (65 years and over) had significantly greater vitamin D intakes compared to other age categories (3.28 ± 2.27 μg/day), this age group observed the smallest relative increase from the dietary modeling scenarios (14.3%) (Table 2). This may be owed to fish and fish dishes also substantially contributing to vitamin D intakes in older adults (Supplementary Material). The greatest relative change was observed amongst 11–18 years, where 200% vitamin D biofortification of pork and pork products would result in a 25.2% increase in mean daily vitamin D intakes. The range of vitamin D intakes from 50, 100, 150, and 200% scenarios were <0.01–22.42, <0.01–22.66, <0.01–22.90, and <0.01–23.14 μg/day, respectively. In general, dietary modeling showed a significant difference between each age cohort, with the exception of 4–10 years and 11–18 years (*p* > 0.05). Significant increases in vitamin D daily intakes were evident from current baseline values for each of the four modeled changes (*p* < 0.05).

**Table 2:** Theoretical mean vitamin D intakes (μg/day), split by gender and age ranges, from diet alone of UK population in response to varying increases in vitamin D concentration of pork and pork products (*n* = 13,350).

**Population**		**Vitamin D intake (μg/day)**				
		**Vitamin D increases in pork**				
		**0%**	**50%**	**100%**	**150%**	**200%**
**All**	Mean ± SD	2.47 ± 1.83^a^	2.59 ± 1.86^b^	2.72 ± 1.90^c^	2.84 ± 1.94^d^	2.96 ± 2.00^e^
*n* = 13,350	Relative change (%)	N/A	4.9	10.1	15.0	19.8
	Maximum (μg/day)	22.19	22.42	22.66	22.90	23.14
**Male**	Mean ± SD	2.66 ± 1.99^a^	2.81 ± 2.03^b^	2.96 ± 2.07^c^	3.11 ± 2.13^d^	3.26 ± 2.21^e^
*n* = 6,161	Relative change (%)	N/A	5.6	11.3	16.9	22.6
**Female** ^†^	Mean ± SD	2.30 ± 1.66^a^	2.40 ± 1.68^b^	2.51 ± 1.70^c^	2.61 ± 1.73^d^	2.71 ± 1.77^e^
*n* = 7,189	Relative change (%)	N/A	4.3	9.1	13.5	17.8
**1.5–3 y**	Mean ± SD	1.97 ± 1.90^a,z^	2.05 ± 1.90^b,z^	2.12 ± 1.91^c,z^	2.20 ± 1.93^d,z^	2.28 ± 1.94^e,z^
*n* = 1,173	Relative change (%)	N/A	4.1	7.6	11.7	15.7
**4–10 y**	Mean ± SD	2.01 ± 1.19^a,y^	2.12 ± 1.22^b,y^	2.23 ± 1.26^c,y^	2.35 ± 1.30^d,y^	2.46 ± 1.36^e,y^
*n* = 2,554	Relative change (%)	N/A	5.5	10.9	16.9	22.4
**11–18 y**	Mean ± SD	2.14 ± 1.38^a,y^	2.27 ± 1.42^b,y^	2.41 ± 1.48^c,y^	2.54 ± 1.55^d,y^	2.68 ± 1.63^e,y^
*n* = 2,821	Relative change (%)	N/A	6.1	12.6	18.7	25.2
**19–64 y**	Mean ± SD	2.74 ± 1.99^a,x^	2.88 ± 2.02^b,x^	3.01 ± 2.07^c,x^	3.14 ± 2.13^d,x^	3.28 ± 2.19^e,x^
*n* = 5,223	Relative change (%)	N/A	5.1	9.9	14.6	19.7
**65+ y**	Mean ± SD	3.28 ± 2.27^a,w^	3.40 ± 2.29^b,w^	3.51 ± 2.31^c,w^	3.63 ± 2.34^d,w^	3.75 ± 2.37^e,w^
*n* = 1,579	Relative change (%)	N/A	3.7	7.0	10.7	14.3

*Data is presented as mean ± standard deviation, unless otherwise specified. Values not sharing a common superscript letter in rows (a, b, c, d, e) are significantly different (p < 0.001) between modeling scenarios; one-way repeated measures ANOVA using log transformed data*.

† *Denotes all mean intakes are significantly different between males and females; independent t-test. Values not sharing a common superscript letter in columns (z, y, x, w) are significantly different (p < 0.05) between age ranges of the same modeling scenario; one-way ANOVA with Tukey test using log transformed data. UK, United Kingdom; n, number of participants; SD, standard deviation; y, years; N/A, not applicable*.

### 25(OH)D Concentrations

**Figure 3:**
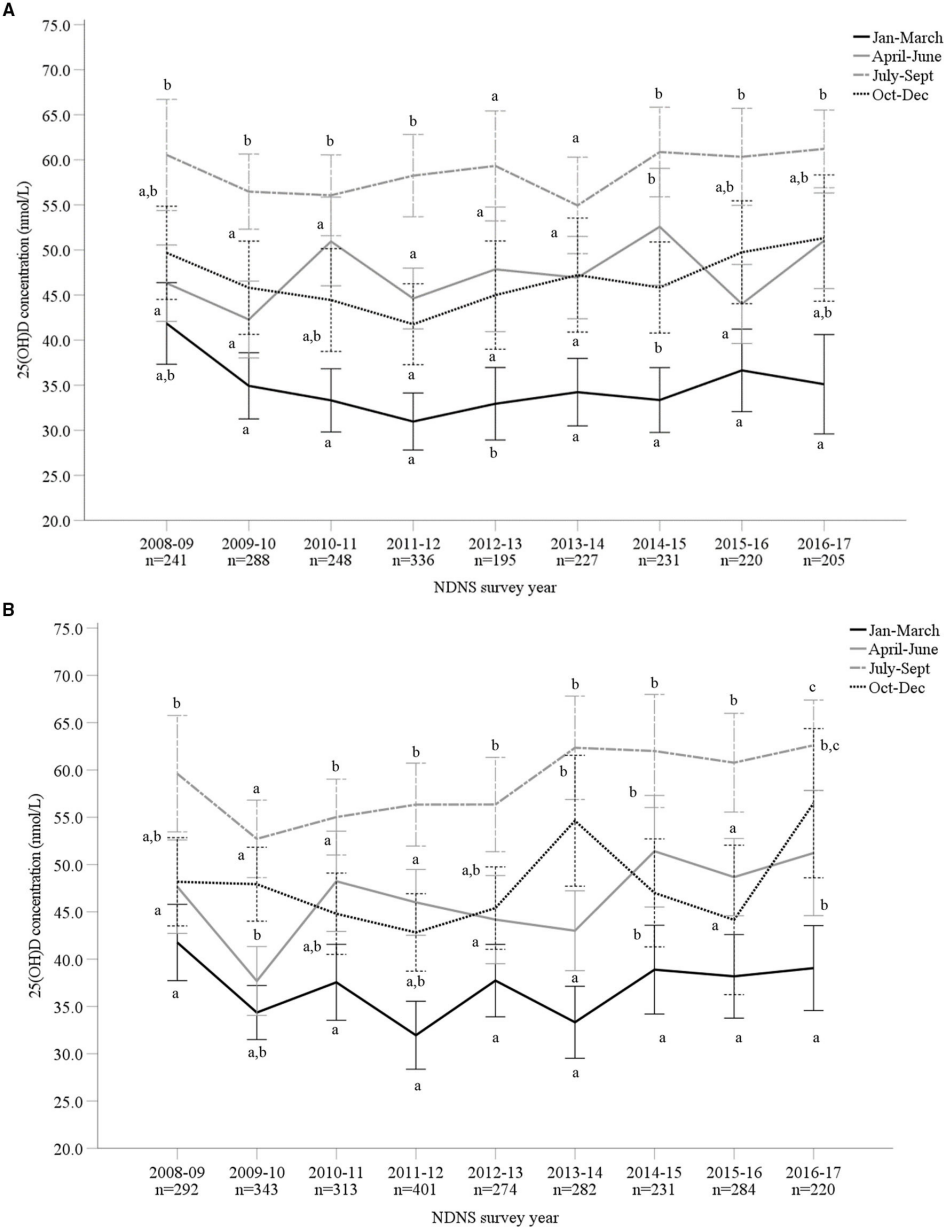
**(A,B)** Vitamin D status [25-hydroxyvitamin D (25(OH)D) nmol/L] of male (**A**; *n* = 2,191) and female (**B**; *n* = 2,640) adults aged 19–64 years from Years 1–9 (2008–2017) of the UK National Diet and Nutrition Survey (NDNS; total *n* = 4,831). Data is presented as mean (95% CI). Values not sharing a common superscript letter (a, b, c) are significantly different (*p* < 0.05) between seasons in each survey year; one-way ANOVA and *post-hoc* (Tukey) tests. 25(OH)D concentration data from standardized liquid chromatography coupled to tandem mass spectrometry (LC-MS/MS). UK, United Kingdom; *n*, number of participants; CI, confidence interval.

**Figure 4:**
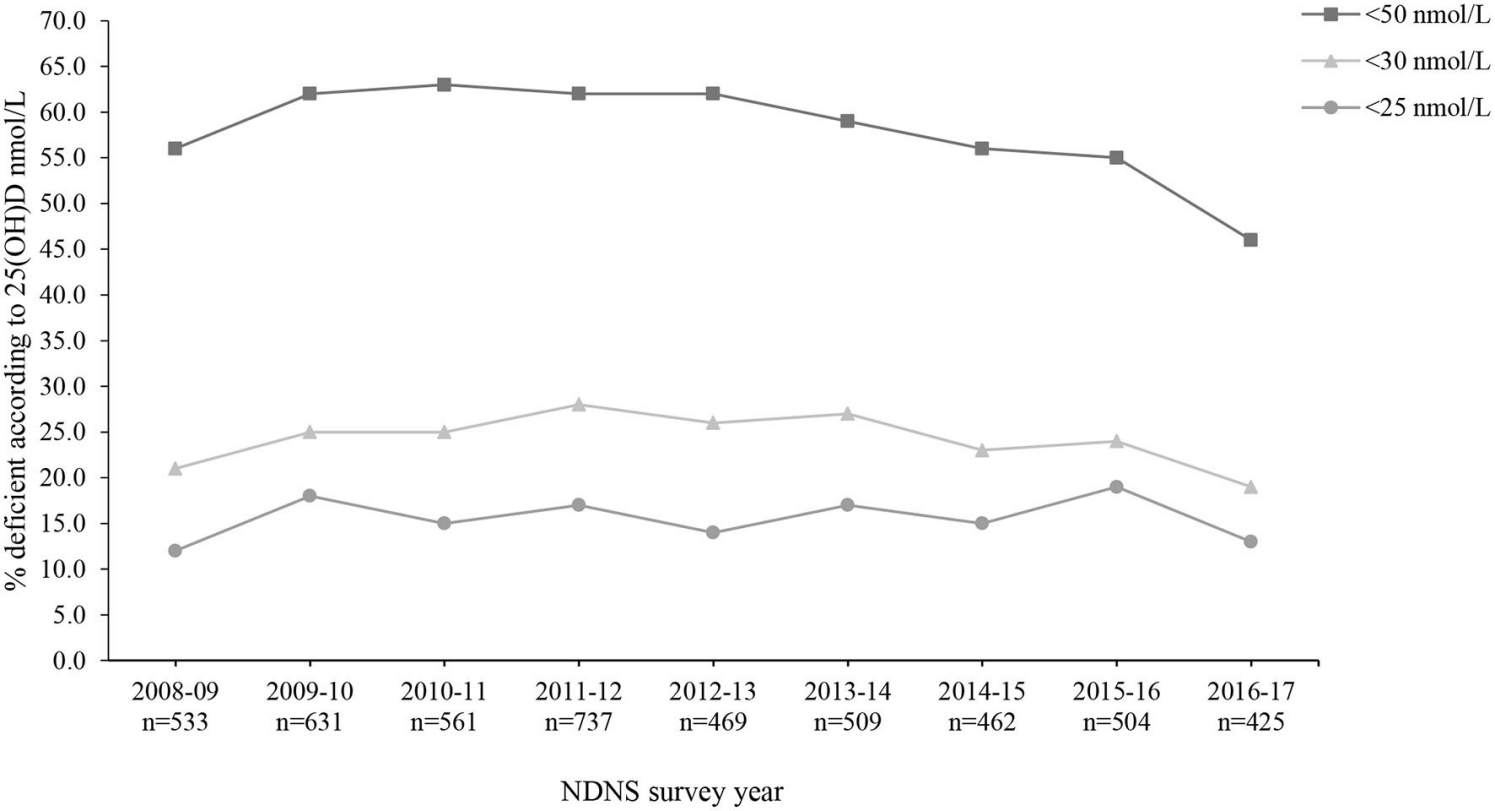
Percentage (%) of participants from the UK National Diet and Nutrition Survey (NDNS) classified as vitamin D deficient based on The Endocrine Society and EFSA (<50 nmol/L), US Institute of Medicine (<30 nmol/L) and SACN (<25 nmol/L) cut-off values for 25(OH)D concentrations (*n* = 4,831). UK, United Kingdom; EFSA, European Food Safety Authority; US, United States; SACN, Scientific Advisory Committee on Nutrition (UK); 25(OH)D, 25-hydroxyvitamin D; *n*, number of participants who provided blood sample.

Overall, mean 25(OH)D concentrations across all 9 years were 46.8 ± 21.2 nmol/L (M 46.5 ± 20.6 nmol/L and F 47.1 ± 21.6 nmol/L). The lowest and highest mean 25(OH)D concentrations were observed in 2009–2010 (44.5 ± 19.4 nmol/L) and 2016–2017 (51.7 ± 22.5 nmol/L), respectively. The most recent data (for 2016/17) reported significantly increased 25(OH)D concentrations compared to 2009–2014 (*p* < 0.05). However, when split by age categories, those aged 1.5–3 years and 11–18 years reported no significant difference across all 9 survey years (*p* > 0.05) (Supplementary Material). For both males and females, vitamin D status significantly varied across seasons, except 2013/14 in males, with the highest concentration observed during late summer months (July to September) and lowest in late winter (January to March) (Figures 3A,B; Supplementary Material). Figure 4 presents the percentage of participants classified as vitamin D deficient based on different cut-off levels. In 2016/17, when including all age groups, 45.6, 19.3, and 13.2% presented 25(OH)D concentrations deemed insufficient (<50, 30, and 25 nmol/L, respectively; Supplementary Material). Across all nine survey years, 15.6% of participants had 25(OH)D concentrations <25 nmol/L (mean 18.9 ± 5.1 nmol/L) (4). This increased to 24.4 and 58.5% when considering <30 nmol/L (mean 22.0 ± 5.8 nmol/L) and <50 nmol/L (mean 32.3 ± 10.5 nmol/L) as the deficiency threshold classified by the U.S. Institute of Medicine and The Endocrine Society, respectively (5, 7).

## Discussion

For almost a decade, mean vitamin D intakes have remained suboptimal and stagnant in the UK. Only 17% of adults (19–64 years) reported supplementing with vitamin D in 2016/17 and a larger proportion of females take a vitamin D supplement compared to men (20 vs. 13%) (40) thus, it would appear that there has been limited implementation following updated vitamin D supplement advice in 2016 (4). The COVID-19 pandemic, however, has heightened public interest and awareness of the importance of vitamin D (41) and it may be postulated that this has resulted in a greater uptake in supplementation which may be reflected in future data. Nevertheless, the current findings confirm that vitamin D intakes are unacceptably low and strongly supports the need for mandatory biofortification as an additional food-based solution to offer a diet-focused approach in alleviating vitamin D deficiency.

The current paper, for the first time, demonstrates that increasing the vitamin D content in pork and pork products, without altering consumers habitual diet, resulted in significant, albeit modest, increases to the vitamin D intakes within the UK population. It is important to be cognizant that this theoretical change was based on achievable increases in vitamin D that can be reasonably expected using natural biofortification, for example UVB radiation and enriched animal feed, rather than traditional exogenous fortification (38, 39). Previous research has theorized the potential impact of vitamin D fortification in ready-to-eat cereals (RTEC), milk, bread, plain yogurt, cheese, eggs, crackers, and wheat flour (17–19, 30, 31). In these studies, as expected, predicted exogenous fortification produced higher theoretical intakes compared to those reported within the present study. Fortification of semi-skimmed cows' milks theoretically increased median vitamin D intakes from 2.3 to 6.1 μg/day (17) whilst fortifying both milk and bread resulted in ~70% of Irish individuals meeting the recommended 10 μg/day (19). Notably, endogenous vitamin D biofortification offers an increased challenge as greater natural inter and intra-variability exists in end-point vitamin D concentrations in meat, compared to traditional fortification practices whereby there is rigid control on the quantity of vitamer being added to the foodstuff during processing. However, biofortification may be perceived as a more natural food-based strategy and presents vast opportunities for both the food industry and populations.

Additionally, improvements within the current paper were only modeled based on altering only one food type (pork) within a main food group (meat). Even greater improvements would be expected if this practice was implemented across a wider animal food portfolio to account for dietary diversity and include, for example, chicken, beef and eggs, of which on-farm evidence provides proof of concept (24, 42–44). Regardless of the biofortification food carrier, it would be short-sighted and ill-advised to focus on a single commodity as this would undoubtedly exclude a proportion of the population who identify as low or non-consumers. Of note, owing to the rise in vegetarian and vegan dietary trends, motivated by environmental and health concerns (45), coupled with the prevalence of religious groups vulnerable to vitamin D deficiency such as veiled Moslem women living in Europe, additional plant-based strategies must also be considered to maximize the benefits of biofortification. Human randomized controlled trials (RCTs) have investigated the efficacy of biofortified mushrooms and bread baked with UV-treated yeast. Whilst bread had poor bioavailability, mushrooms may be an alternative biofortification food vehicle to increase 25(OH)D_2_ concentrations amongst non-meat consumers (24). Future research should further explore bioavailability of UV-treated yeast to confirm these findings. Despite vitamin D_3_ being considered more effective than vitamin D_2_ (46, 47), and 25(OH)D_2_ often increasing at the apparent expense of 25(OH)D_3_, for vegans and vegetarians who may have limited vitamin D_3_ in their diet, total 25(OH)D concentrations should increase with vitamin D_2_-biofortified products. Owing to limited viable non-animal biofortified foods, vegetarian and vegan consumers may be more likely to benefit from fortified foods such as breakfast cereals (18, 48), wheat flour/bread (19, 31, 48, 49) and fruit juice (48, 50). Nonetheless, meat remains a popular staple component for a large proportion of the UK population (40). Therefore, it is advantageous for those who choose to include pork and pork products in their diet to have access to high-quality, nutrient-dense meat to aid in reaching nutritional recommendations. The marketed nutritional benefit of vitamin D biofortified pork, however, should ensure the avoidance of a “halo effect” or positivity bias (51–53), whereby the enhanced micronutrient content may encourage consumers to contradict the recommendation to reduce red and processed meat intake.

Future vitamin D dietary modeling should explore the proportion of population below the RNI and exceeding the tolerable upper intake level (UL) in various scenarios using both animal and plant-based foods, combined with the inclusion and exclusion of varying supplemental intakes. This evaluation will inform how to achieve the goal of having nearly the entire population above the recommended intake without exceeding the UL. Future dietary modeling research may also explore environmental or individual factors associated with suboptimal 25(OH)D concentrations such as educational status, socioeconomic status and genetics (54, 55). Importantly, there is a need to ensure those of lower socioeconomic status are not further disadvantaged by biofortified products being a premium price or having an image of exclusivity. Moreover, prior to the implementation of vitamin D biofortification, there should be a thorough assessment of its efficacy, feasibility, production costs for farmer and food industry, as well as consideration for regulatory aspects (48).

Notably, biofortification would only be effective if it is part of a mandatory vitamin D fortification program by the government, with both strategies recognized as the only measure to improve vitamin D status in the general population. The United States, Canada and Finland have effectively implemented mass vitamin D food fortification policies (16, 22, 23) which should act as a benchmark for the UK. If enforced, revision of supplement guidance may be required to assess risk of toxicity and reduce the likelihood of exceeding the 50–100 μg/day (2,000–4,000 IU/day) UL for adults and children (4, 48). Within the current modeling scenario however, safety concerns are low and no participant exceeded the UL following vitamin D biofortification of pork and pork products. This differs to supplement intake which can pose risk of toxicity depending on the quantity and frequency of consumption. Importantly, pork meat biofortification holds a degree of biological regulatory control, compared to traditional fortification. Similar to humans, in UVB-exposed pigs the CYP24A1 enzyme is induced which acts as a feedback mechanism to prevent vitamin D toxicity (56).

Owing to stable vitamin D intakes, it is unsurprising that, in general, 25(OH)D concentrations also reflected this outcome. Considering all age groups, some significant increases in 25(OH)D concentrations were observed in 2016/17 compared to earlier years which is encouraging; however, this was not consistent when split by age groups. Nevertheless, as hypothesized, significant changes were observed between seasons owed to variation in UVB radiation during warmer months (April to September). Vitamin D is a fat-soluble prohormone and therefore some bodily stores remain prior to the expected wintertime nadir, explaining the gradual decline to the lowest status observed in early spring. The consistency of insufficient vitamin D status further highlights the need for additional strategies. Owing to such seasonal variations, calculations could be performed based on the predicted timeline for on-farm UV biofortification duration, slaughter and pork processing to identify pork sold during September to April and thus, sector biofortification to the seasons where suboptimal status is most prevalent. However, this may present industrial challenges and complications which may not be realistic in the commercially relevant or widescale context. If mandatory pork biofortification was implemented throughout the year, and at the optimized level (200% increase in vitamin D content), the contribution of cutaneous synthesis during summer months may subsequently decrease to account for greater dietary intakes. Priority should first focus on increasing year-round vitamin D status in the majority of the UK population by a combination of biofortification, fortification and supplemental strategies.

NDNS provides critically important national food intake data however, it is not without limitations. Food diaries, used within the current study, are inherently flawed owing to high prevalence of underreporting. Healthy or unhealthy bias can be present (57) and is often associated with age, body mass index (BMI, kg/m^2^), socioeconomic status and ethnicity (58). Results from doubly labeled water (DLW), a well-recognized method to measure energy expenditure in free-living individuals, suggest that energy intake reported by participants has been underreported (energy intake: total energy expenditure = 0.73). A detailed overview of DLW results from NDNS subsamples have been described elsewhere (35).

Future research should model 25(OH)D concentrations combined with total vitamin D (vitamin D_3_ + vitamin D_2_). Results may otherwise under-represent the true impact of biofortified pork, with an even greater increase hypothesized if 25(OH)D was independently considered. This is owed to the natural presence of 25(OH)D in pork meat (59, 60), further increases following biofortification practices (24, 38) and suggestions this metabolite may be five times more potent than parental vitamin D to increase circulating 25(OH)D concentrations (25, 61). Although changes to 25(OH)D concentrations were not within the scope of the present study, depending upon absorptive capacity and baseline status, an increase of 1 μg of vitamin D_3_ has been reported to equate to an approximate increase of 0.7–1.0 nmol/L of serum 25(OH)D concentrations (62). Undoubtedly, there is a need for acute and chronic human RCTs to explore the bioavailability, bioaccessibility and real-life application of the modeling scenarios within this study to confirm the impact of vitamin D biofortified pork meat on circulating 25(OH)D concentrations; thus, potentially reducing musculoskeletal risks or suboptimal immune functioning.

## Conclusion

Evidently, there is no panacea for hypovitaminosis D; rather, an integrated strategy is urgently required to reduce prevalence rates. Mandatory biofortification may offer an easily implemented strategy to help bridge the gap between current vitamin D intakes and those recommended by government guidelines in order to limit the risk of musculoskeletal diseases. As meat and meat products provide a sizeable contribution to vitamin D intakes in UK diets, even without biofortification, it is sensical to improve the vitamin D content within these food groups to benefit the UK population and, in particular, at-risk subgroups and supplement non-users. Due to the prominence of meat in UK diets, a lower resistance to uptake would be anticipated and potentially allow for greater impact. Nevertheless, a combined approach using a range of biofortified food products is required to ensure the number of non-consumers is limited and the widest proportion of the population can be reached, particularly those susceptible to lower 25(OH)D concentrations. With a clear need to prioritize and strengthen initiatives which will help in alleviating vitamin D deficiency, mandatory biofortification of pork may offer a modest, yet vitally important contribution to increasing intakes, particularly in adolescents.

## Data Availability Statement

Publicly available datasets were analyzed in this study. This data can be found here: NatCen Social Research and MRC Elsie Widdowson Laboratory (37).

## Author Contributions

HN, CG, and LP designed the research. HN combined the database, performed analyses, and prepared the manuscript. All authors contributed to interpreting the results, read and approved the final manuscript.

## Funding

This work was funded as part of a Department for the Economy (DfE) Co-operative Awards in Science and Technology (CAST) PhD studentship, supported by Devenish Nutrition Limited.

## Conflict of Interest

EJM was employed by the industrial partner Devenish Nutrition Ltd. The authors declare that this study received funding from Devenish Nutrition Ltd., in the form of a CAST PhD Studentship awarded to the lead author. The funder had the following involvement in the study: review and approval of the final manuscript. The remaining authors declare that the research was conducted in the absence of any commercial or financial relationships that could be construed as a potential conflict of interest.

## Publisher's Note

All claims expressed in this article are solely those of the authors and do not necessarily represent those of their affiliated organizations, or those of the publisher, the editors and the reviewers. Any product that may be evaluated in this article, or claim that may be made by its manufacturer, is not guaranteed or endorsed by the publisher.
