# Supplementary material for: Vitamin D Biofortification of Pork May Offer a Food-Based Strategy to Increase Vitamin D Intakes in the UK Population
Source: Front Nutr. 2021 Dec 3;8:777364. doi: 10.3389/fnut.2021.777364 (PMC8679618; doi:10.3389/fnut.2021.777364)
Supplement: Supplementary file 1 [file Data_Sheet_1.pdf]

Supplemental Material

Supplemental Table 1. Food name and code from UK National Diet and Nutrition Survey (NDNS) selected for vitamin D-enriched pork dietary modeling syntax.

Main Food Group Code	Main Food Group Description	Food Number	Food Name
22	Bacon and Ham	10263	Bacon and cheese grills
		901	Bacon collar joint boiled lean and fat
		9725	Bacon collar joint roasted
		8232	Bacon collar smoked boiled L&F
		903	Bacon gammon joint boiled lean and fat
		904	Bacon gammon joint boiled lean only
		8233	Bacon gammon joint smoked boiled L&F
		906	Bacon gammon rashers grilled lean and fat
		907	Bacon gammon rashers grilled lean only
		8234	Bacon joint smoked boiled lean only
		5407	Bacon lean grilled dry fried cut unspecified
		910	Bacon rashers back fried lean and fat
		914	Bacon rashers back grilled lean and fat
		9464	Bacon rashers back not smoked grilled extra trim
		8247	Bacon rashers cut unspec smoked grilled lean and fat
		909	Bacon rashers fried lean and fat
		911	Bacon rashers middle fried lean and fat
		915	Bacon rashers middle grilled lean & fat
		8245	Bacon rashers other cut smoked grilled lean& fat
		9412	Bacon rashers red fat+salt not smoked grilled
		912	Bacon rashers streaky fried lean and fat
		916	Bacon rashers streaky grilled lean and fat
		9413	Bacon steaks chops loin not smoked grilled
		11196	Cooked smoked bacon strips e.g. Tesco
		9754	Gammon steak fried in lard
		6924	Glazed baked gammon
		1236	Ham in natural juice canned
		8697	Ham low fat e.g. delight
		9382	Ham no added water, not smoked
		1039	Ham not smoked
		1040	Ham smoked
		8235	Ham smoked deli or butchers
		8236	Ham smoked vacuum packed
		9508	Ham unspecified not smoked not canned
		9509	Ham unspecified smoked
		9381	ham with added water not smoked
		10398	M&S smoked bacon and cheese crispbakes
		8089	Parma ham
		9385	Pork shoulder
		913	Rashers any other cut grilled lean and fat
		8237	Rashers back smoked fried L&F

		8238	Rashers back smoked grilled L&F
		8246	Rashers bacon smoked fried lean and fat
		908	Rashers cut unspec not smoked grilled lean and fat
		8239	Rashers gammon smoked grilled L&F
		8241	Rashers middle smoked fried lean and fat
		8242	Rashers middle smoked grilled L&F
		8240	Rashers smoked gammon grilled lean only
		8244	Rashers smoked streaky grilled lean and fat
		8243	Rashers streaky smoked fried lean and fat
		9410	Smoked bacon back extra trim grilled or dry fried
		9414	Smoked bacon steaks/chops
		9384	Smoked ham no added water any cut
		9383	Smoked ham with added water any cut
		9411	Smoked bacon grilled or dry fried red salt/fat
25	Pork and dishes	5452	Bacon burger
		9786	Bacon burger with cheese
		6156	Chinese dumplings
		9460	Diced pork stewed lean only
		9462	Fillet (tinderloin) grilled lean
		5249	Lasagne made with pork
		9461	Minced pork stewed lean + fat
		8012	Pork and beef meatballs oven baked or grilled
		1020	Pork belly rashers slices roast lean & fat no bone
		1022	Pork belly rashers stewed L&F
		9873	Pork burgers made with extra lean pork
		5324	Pork casserole made with canned cook-in-sauce
		5695	Pork casserole with potatoes carrots and swede
		10845	Pork chow mein
		9452	Pork chump chops steak grilled lean + fat no bone
		9454	Pork chump chops steaks grilled lean only no bone
		9402	Pork crackling cooked
		9490	Pork diced raw lean and fat
		3808	Pork diced stewed lean and fat
		5735	Pork escalope pork in E&C fried in vegetable oil
		9448	Pork hand or spring joint roasted lean and fat
		9449	Pork hand or spring joint roasted lean only
		1041	Pork L&F gravy carrot onion
		1042	Pork lean gravy carrot onion
		1032	Pork leg joint knuckle fillet roast lean and fat
		1033	Pork leg joint knuckle fillet roast lean only
		9458	Pork leg steaks chops grilled lean only no bone
		9456	Pork leg steaks chops grilled lean+fat no bone
		1028	Pork loin chop with kidney grilled lean no bone
		1027	Pork loin chops steaks grilled lean & fat + bone
		1026	Pork loin chops steaks grilled lean & fat no bone
		1024	Pork loin chops steaks grilled lean only no bone
		1025	Pork loin chops steaks grilled lean only with bone
		9451	Pork loin joint roasted lean only

		9450	Pork loin joint roasted lean+fat
		10208	Pork meatballs canned in tomato sauce or gravy
		6009	Pork meatballs homemade
		6168	Pork meatballs in tomato sauce
		1352	Pork roast dinner frozen ready meal
		8249	Pork roast roll cooked bernard matthews
		5323	Pork roast slices
		9444	Pork spare rib chops braised lean & fat no bone
		9446	Pork spare rib chops braised lean only no bone
		9443	Pork spare rib joint pot roasted lean only
		9442	Pork spare rib shoulder joint braised lean & fat
		9463	Pork spare ribs belly grilled lean & fat
		9862	Pork steak casserole
		3181	Pork steaks or shank with honey & mustard sauce purchased
		1331	Spare ribs in barbecue sauce no bones
		1355	Spare ribs, barbecue style, e.g. takeaway, with bones
		5561	Stovies (pork & potato fried in veg oil)
		6051	Sweet and sour pork code 6051
		9763	Sweet and sour pork frozen ready meal no rice
		1358	Sweet and sour pork, battered with/without sauce
30	Sausages	10155	Chorizo
		1271	Frankfurter
		1272	Frankfurter canned
		5308	Frankfurter in a bun with ketchup onions & must
		1273	Polony
		10749	Pork and chicken hotdogs with vegetables canned
		8268	Pork sausage smoked fried
		8269	Pork sausage smoked grilled
		4008	Pork sausagemeat, coated in breadcrumbs, grilled or oven baked, e.g. 'walls balls'
		3784	Pork sausages, very low fat, grilled
		1284	Sausage in batter fry blended
		1288	Sausage in batter fry comm oil
		8772	Sausage meat stuffing
		7784	Sausages economy fried
		6243	Sausages in batter grilled eg walls wall banger
		7789	Sausages pork & beef skinless grilled
		7788	Sausages pork and beef fried
		7786	Sausages pork skinless fried
		7787	Sausages pork skinless grilled
		7792	Sausages premium pork fried
		1279	Sausages, pork, fried
		1283	Sausages, low fat, pork, grilled
		1282	Sausages, pork and beef, grilled, fried
		7785	Sausages, pork, economy, grilled
		1280	Sausages, pork, grilled
		7793	Sausages, premium pork, grilled
		1290	Saveloy

		10470	Toad-in-the-hole ready meals purchased
		5326	Toad-in-the-hole made with pork sausages & ssmilk
		10347	Weight watchers sausages in cider gravy with vegetable mash ready meal
31	Meat pies and pastries	10289	Beef and pork pie in shortcrust pastry retail
		10264	Chicken and bacon pies purchased
		3225	Chicken, bacon, mushroom & cream pie
		6752	Ham and mozzarella pastry
		1303	Pork and egg pie
		7796	Pork pie buffet
		1304	Pork pie individual
		1305	Pork pie sliced
		3193	Pork sausage snack bar
		1306	Sausage roll flaky pastry
		8071	Sausage roll flaky pastry purchased
		1307	Sausage roll shortcrust pastry
		1308	Sausage roll shortcrust pastry purchased
		10350	Sausage rolls purchased reduced fat
		9859	Spring roll with meat and vege
		1351	Spring roll with meat filling
32	Other meat and meat products	1315	Back and egg in a muffin, bagel or roll takeaway
		3819	Black pudding batter commercial fried
		1247	Black pudding boiled
		1248	Black pudding fried
		3819	Black pudding in batter takeaway
		4108	Chopped ham and pork with egg
		1250	Faggots in gravy ready meal
		1337	Garlic sausage
		1237	Ham and pork chopped canned
		1255	Haslet
		1178	Kidney pigs fried or grilled
		1338	Luncheon meat not canned
		1239	Luncheon meat pork canned
		1332	Meat chop suey pork beef lamb
		8267	Pepperami
		9590	Pork tongue
		1274	Salami
		10067	Sausage and egg in a muffin, bagel or roll, takeaway
		5623	Spam fritters
		8694	Turkey and pork luncheon meat e.g. Sainsburys billy bear
		1263	White pudding

Information gained from ‘Food Level Dietary Data’ files available from the UK Data Archives (NatCen, University of Essex, Colchester, Essex, UK).

Supplemental Table 2. Percentage contribution of food groups to mean vitamin D intake (µg/day) in UK diets, split by age ranges.

	NDNS Years 1-11 combined (2008-2019)						
**Food group**	**All ages**	**1.5-3 y**	**4-10 y**	**11-18 y**	**19-64 y**	**65-74 y**	**75+ y**
Meat & meat products (%)	25	18	23	32	29	23	22
Cereals & cereal products (%)	18	19	26	23	14	12	17
Fat spreads (%)	15	15	16	15	14	14	15
Fish & fish dishes (%)	14	7	8	9	16	24	20
Eggs & egg dishes (%)	14	11	10	11	17	18	15
Milk & milk products (%)	11	27	14	7	6	5	6
Miscellaneous* (%)	4	4	4	3	4	3	4

*Includes vegetables and potatoes, savoury snacks, nuts and seeds, fruit, sugar, preserves and confectionery, non-alcoholic beverages, and alcoholic beverages. UK, United Kingdom; NDNS, National Diet and Nutrition Survey; y, years. Data obtained from NDNS: results from years 9 to 11 (combined) – data tables. Available from www.gov.uk.

Supplemental Table 3. Vitamin D intake (µg/day) from diet alone and in combination with supplements from Years 1-9 (2008-2017) of the UK National Diet and Nutrition Survey (NDNS).

**Survey Year**	**Vitamin D intake (µg/day)**					
	**Diet only**			**Diet & Supplements**		
	**All (*n*=13,350)**	**Male (*n*=6,161)**	**Female (*n*=7,189)**	**All (*n*=13,350)**	**Male (*n*=6,161)**	**Female (*n*=7,189)**
**2008-2017 (All Years)** *n*=13,350	2.47 ± 1.83	2.66 ± 1.99	2.30 ± 1.66*	3.46 ± 6.32	3.42 ± 4.42	3.50 ± 7.59*
**2008-2009 (Year 1)** *n*=1,646	2.49 ±1.79	2.70 ± 2.05	2.32 ± 1.52*	3.14 ± 2.78	3.37 ± 2.92	2.95 ± 2.63*
**2009-2010 (Year 2)** *n*=1,669	2.42 ± 1.90	2.58 ± 2.17	2.27 ± 1.62*	3.12 ± 3.02	3.14 ± 3.03	3.10 ± 3.02
**2010-2011 (Year 3)** *n*=1,565	2.38 ± 1.66	2.59 ± 1.78	2.20 ± 1.53*	3.05 ± 3.81	3.10 ± 3.57	3.01 ± 4.01*
**2011-2012 (Year 4)** *n*=1,948	2.56 ± 1.87	2.81 ± 2.00	2.35 ± 1.73*	3.35 ± 3.36	3.44 ± 2.96	3.27 ± 3.66*
**2012-2013 (Year 5)** *n*=1,197	2.55 ± 1.99	2.84 ± 2.26	2.30 ± 1.71*	3.52 ± 5.72	3.70 ± 3.82	3.37 ± 6.90*
**2013-2014 (Year 6)** *n*=1,349	2.46 ± 1.84	2.58 ± 1.90	2.36 ± 1.79*	3.78 ± 9.71	3.37 ± 5.86	4.11 ± 11.94
**2014-2015 (Year 7)** *n*=1,353	2.42 ± 1.80	2.60 ± 1.97	2.25 ± 1.61*	3.56 ± 7.99	3.40 ± 4.16	3.71 ± 10.35
**2015-2016 (Year 8)** *n*=1,370	2.44 ± 1.74	2.62 ± 1.93	2.29 ± 1.56*	3.68 ± 6.89	3.69 ± 7.61	3.68 ± 6.23
**2016-2017 (Year 9)** *n*=1,253	2.51 ± 1.83	2.61 ± 1.78	2.42 ± 1.87*	4.29 ± 10.66	3.73 ± 4.79	4.79 ± 13.92

Data is presented as mean ± standard deviation. *Denotes significant difference ($p < 0.05$) within rows between male and female participants (comparison within either 'diet only' or 'diet & supplements'); independent samples *t* test using log transformed data. No significant difference ($p > 0.05$) within column between survey years; one-way ANOVA and *post hoc* (Tukey) tests using log transformed data. UK, United Kingdom; *n*, number of participants; y, years.

Supplemental Table 4. Vitamin D intake (µg/day) from diet alone and in combination with supplements, split by age categories, from Years 1-9 (2008-2017) of the UK National Diet and Nutrition Survey (total *n*=13,350).

**Survey Year**	**Vitamin D intake (µg/day)**									
	**Diet only**					**Diet & Supplements**				
	**1.5-3 y**	**4-10 y**	**11-18 y**	**19-64 y**	**65+ y**	**1.5-3 y**	**4-10 y**	**11-18 y**	**19-64 y**	**65+ y**
**2008-2017 (All Years)** *n*=13,350	1.97 ± 1.90^{a}	2.01 ± 1.19^{b}	2.14 ± 1.38^{b}	2.74 ± 1.99^{c}	3.28 ± 2.27^{d}	2.62 ± 2.76^{a}	2.76 ± 4.16^{b}	2.49 ± 5.22^{a}	3.99 ± 8.11^{c}	5.19 ± 5.63^{d}
**2008-2009 (Year 1)** *n*=1,646	1.83 ± 1.83^{a,z}	1.97 ± 1.03^{b,z,y}	2.19 ± 1.26^{b,z}	2.87 ± 1.94^{c,z}	3.37 ± 2.49^{d,z}	2.30 ± 2.21^{a,z,y}	2.54 ± 1.92^{b,z}	2.34 ± 1.48^{a,b,z}	3.68 ± 3.17^{c,z}	4.73 ± 3.82^{d,z}
**2009-2010 (Year 2)** *n*=1,669	1.80 ±1.93^{a,z}	1.87 ± 0.95^{b,z,y}	2.09 ± 1.21^{b,z}	2.83 ± 2.30^{c,z,y}	3.12 ± 2.20^{c,z}	2.16 ± 2.33^{a,z}	2.48 ± 1.82^{b,z}	2.41 ± 1.75^{b,z}	3.67 ± 3.48^{c,z}	4.63 ± 4.36^{d,z}
**2010-2011 (Year 3)** *n*=1,565	1.93 ± 1.56^{a,z}	1.99 ± 1.22^{a,z,y}	2.09 ± 1.25^{a,z}	2.59 ± 1.75^{b,z,y}	3.38 ± 2.28^{c,z}	2.09 ± 1.95^{a,z,y}	2.80 ± 5.16^{b,z}	2.26 ± 1.63^{a,b,z}	3.31 ± 3.84^{c,z}	5.19 ± 4.75^{d,z}
**2011-2012 (Year 4)** *n*=1,948	2.16 ± 2.04^{a,z}	2.06 ± 1.23^{a,z,y}	2.08 ± 1.36^{a,z}	2.83 ± 1.97^{b,z,y}	3.40 ± 2.39^{c,z}	2.56 ± 2.55^{a,z,y}	2.58 ± 2.04^{a,z}	2.32 ± 1.80^{a,z}	3.70 ± 3.62^{b,z}	5.44 ± 4.93^{c,z}
**2012-2013 (Year 5)** *n*=1,197	2.23 ± 2.45^{a,z}	2.03 ± 1.39^{a,b,z,y}	2.34 ± 1.74^{b,c,z}	2.82 ± 2.08^{c,d,z,y}	3.00 ± 2.23^{d,z}	3.25 ± 3.70^{a,z,y}	3.35 ± 10.69^{a,z}	2.62 ± 2.05^{a,z}	3.88 ± 4.39^{b,z}	4.27 ± 3.78^{b,z}
**2013-2014 (Year 6)** *n*=1,349	1.77 ± 1.83^{a,z}	1.98 ± 1.15^{b,c,z,y}	2.22 ± 1.52^{c,d,z}	2.69 ± 2.01^{d,z,y}	3.44 ± 2.21^{e,z}	2.55 ± 3.07^{a,z,y}	2.89 ± 3.46^{a,b,z}	2.57 ± 2.51^{a,z}	4.75 ± 15.16^{b,z}	5.33 ± 5.96^{c,z}
**2014-2015 (Year 7)** *n*=1,353	1.85 ± 1.64^{a,z}	1.95 ± 1.44^{a,b,z}	2.14 ± 1.40^{b,z}	2.64 ± 1.94^{c,z,y}	3.20 ± 2.06^{d,z}	2.63 ± 2.60^{a,z,y}	2.59 ± 2.24^{a,z}	3.27 ± 15.15^{a,z}	3.77 ± 5.19^{b,z}	5.34 ± 5.91^{c,z}
**2015-2016 (Year 8)** *n*=1,370	2.10 ± 1.88^{a,z}	2.05 ± 1.09^{a,z,y}	2.11 ± 1.38^{a,z}	2.61 ± 1.87^{b,y}	3.37 ± 2.21^{c,z}	3.40 ± 3.48^{a,b,y}	2.75 ± 2.30^{a,z}	2.37 ± 2.00^{a,z}	4.42 ± 9.84^{b,z}	5.19 ± 5.91^{c,z}
**2016-2017 (Year 9)** *n*=1,253	2.19 ± 1.89^{a,z}	2.20 ± 1.27^{a,b,y}	2.01 ± 1.37^{a,z}	2.77 ± 1.96^{b,c,z,y}	3.19 ± 2.33^{c,z}	3.16 ± 2.89^{a,b,y}	3.12 ± 2.62^{a,z}	2.52 ± 4.86^{b,z}	5.28 ± 15.65^{a,c,z}	6.50 ± 9.27^{c,z}

Data is presented as mean ± standard deviation. Values not sharing a common superscript letter (a, b, c, d, e) in rows are significantly different ($p < 0.05$) between age groups in the same survey year (comparison within either 'diet only' or 'diet & supplements'); one-way ANOVA and *post hoc* (Tukey) tests using log transformed data. Values not sharing a common superscript letter (z, y) in columns are significantly different ($p < 0.05$) between survey years in the same age group; one-way ANOVA and *post hoc* (Tukey) tests using log transformed data. UK, United Kingdom; *n*, number of participants; y, years.

Supplemental Table 5. Vitamin D status (25-hydroxyvitamin D (25(OH)D) nmol/L) of adults aged 19-64 years from Years 1-9 (2008-2017) of the UK National Diet and Nutrition Survey (total *n*=4,831).

**Survey Year**	**Male (*n*= 2,191)**				**Female (*n*=2,640)**			
	**Jan-March**	**April-June**	**July-Sept**	**Oct-Dec**	**Jan-March**	**April-June**	**July-Sept**	**Oct-Dec**
**2008-2009 (Year 1)** *n*=533	36.13 ± 16.00[a,b]	37.00 ± 22.31[a]	50.40 ± 23.33[b]	43.97 ± 17.10[a,b]	33.84 ± 21.82[a]	40.84 ± 20.70[a]	53.51 ± 24.74[b]	47.84 ± 21.07[a,b]
**2009-2010 (Year 2)** *n*=631	29.85 ± 14.99[a]	29.46 ± 14.44[a]	49.16 ± 24.05[b]	32.83 ± 22.14[a]	30.08 ± 13.98[a,b]	28.47 ± 14.65[b]	43.20 ± 22.73[a]	45.89 ± 20.15[a]
**2010-2011 (Year 3)** *n*=561	29.34 ± 15.33[a]	28.69 ± 13.04[a]	49.76 ± 22.33[b]	42.52 ± 18.60[a,b]	31.90 ± 23.60[a]	35.70 ± 26.40[a]	51.13 ± 23.04[b]	42.46 ± 19.32[a,b]
**2011-2012 (Year 4)** *n*=737	26.15 ± 14.19[a]	23.58 ± 15.79[a]	46.33 ± 22.49[b]	31.30 ± 19.79[a]	32.43 ± 23.65[a]	30.41 ± 15.44[a]	48.61 ± 25.06[b]	37.96 ± 21.09[a,b]
**2012-2013 (Year 5)** *n*=469	26.80 ± 15.30[b]	45.90 ± 26.57[a]	50.23 ± 14.39[a]	44.92 ± 27.89[a]	33.09 ± 17.94[a]	41.32 ± 19.61[a]	54.87 ± 22.58[b]	44.62 ± 21.30[a,b]
**2013-2014 (Year 6)** *n*=509	34.42 ± 21.56[a]	42.80 ± 21.62[a]	49.55 ± 23.32[a]	36.24 ± 18.41[a]	29.26 ± 14.81[a]	36.29 ± 18.00[a]	61.98 ± 24.29[b]	51.75 ± 26.49[b]
**2014-2015 (Year 7)** *n*=462	29.79 ± 11.03[a]	51.45 ± 24.76[b]	54.26 ± 16.28[b]	42.78 ± 18.90[b]	38.78 ± 18.14[a]	51.88 ± 21.68[b]	62.85 ± 25.19[b]	48.82 ± 23.01[b]
**2015-2016 (Year 8)** *n*=504	39.77 ± 18.47[a]	40.82 ± 15.83[a]	56.30 ± 20.63[b]	43.58 ± 19.03[a,b]	37.37 ± 16.94[a]	47.85 ± 20.23[a]	61.35 ± 21.84[b]	47.31 ± 24.28[a]
**2016-2017 (Year 9)** *n*=425	37.17 ± 16.71[a]	48.47 ± 21.37[a,b]	58.19 ± 16.57[b]	47.38 ± 24.19[a,b]	34.78 ± 14.74[a]	51.17 ± 25.72[b]	64.94 ± 19.01[c]	59.76 ± 19.49[b,c]

Data is presented as mean ± standard deviation. Values not sharing a common superscript letter in rows (a, b, c) are significantly different ($p < 0.05$) between seasons in each survey year; one-way ANOVA and *post hoc* (Tukey) tests. 25(OH)D concentration data from standardised liquid chromatography coupled to tandem mass spectrometry (LC-MS/MS). UK, United Kingdom; *n*, number of participants.

Supplemental Table 6. Mean vitamin D status (25-hydroxyvitamin D (25(OH)D) nmol/L) of UK population from 2008-2017 as reported in the UK National Diet and Nutrition Survey (Years 1-9) (total *n*=4,831).

	25(OH)D nmol/L concentration					
**Survey Year**	**All ages**	**1.5-3 y**	**4-10 y**	**11-18 y**	**19-64 y**	**65+ y**
**2008-2009 (Year 1)** *n*=533	48.2 ± 20.8[a,b,c]	62.7 ± 23.3[a]	48.3 ± 19.0[a]	45.5 ± 19.4[a]	48.2 ± 21.0[a,b]	49.7 ± 22.0[a,b]
**2009-2010 (Year 2)** *n*=631	44.5 ± 19.4[a]	51.8 ± 17.0[a]	51.8 ± 14.7[a]	44.5 ± 19.2[a]	43.7 ± 20.3[a]	41.9 ± 18.1[a]
**2010-2011 (Year 3)** *n*=561	45.9 ± 20.3[a,b]	49.9 ± 13.3[a]	54.4 ± 18.9[a,b]	44.2 ± 19.7[a]	45.6 ± 21.3[a,b]	42.2 ± 17.0[a,b]
**2011-2012 (Year 4)** *n*=737	44.8 ± 20.9[a,b]	36.7 ± 13.3[a]	53.0 ± 19.0[a,b]	43.2 ± 20.0[a]	43.9 ± 21.7[a]	45.5 ± 19.8[a,b]
**2012-2013 (Year 5)** *n*=469	45.4 ± 20.4[a,b]	57.1 ± 25.0[a]	49.0 ± 14.6[a]	45.5 ± 21.7[a]	44.6 ± 20.6[a]	44.0 ± 19.5[a,b]
**2013-2014 (Year 6)** *n*=509	46.8 ± 22.3[a,b]	58.6 ± 22.9[a]	54.1 ± 20.0[a,b]	46.5 ± 23.7[a]	45.3 ± 21.7[a,b]	45.9 ± 22.7[a,b]
**2014-2015 (Year 7)** *n*=462	48.6 ± 22.1[b,c]	65.6 ± 21.8[a]	54.3 ± 18.3[a,b]	47.9 ± 22.0[a]	47.6 ± 22.5[a,b]	47.8 ± 22.2[a,b]
**2015-2016 (Year 8)** *n*=504	47.9 ± 21.5[a,b,c]	54.9 ± 23.4[a]	55.9 ± 20.0[a,b]	40.8 ± 22.3[a]	46.8 ± 21.0[a,b]	53.2 ± 20.5[b]
**2016-2017 (Year 9)** *n*=425	51.7 ± 22.5[c]	55.3 ± 26.8[a]	63.2 ± 17.3[b]	43.0 ± 19.7[a]	51.3 ± 22.4[b]	52.5 ± 25.0[b]

Data is presented as mean ± standard deviation. Values not sharing a common superscript letter (a, b, c) in columns are significantly different ($p < 0.05$) between survey years in the same age group; one-way ANOVA and *post hoc* (Tukey) tests. 25(OH)D concentration data from standardised liquid chromatography coupled to tandem mass spectrometry (LC-MS/MS). UK, United Kingdom; *n*, number of participants; y, years.

Supplemental Table 7. Percentage (%) of participants from the UK National Diet and Nutrition Survey classified as vitamin D deficient (<50nmol/L or<30nmol/L), split by age and survey year.

		The Endocrine Society & EFSA				US Institute of Medicine			
**Survey year**	**Total *n****	**% participants 25(OH)D <50 nmol/L (*n* deficient/ *n* total)**				**% participants 25(OH)D <30 nmol/L (*n* deficient/ *n* total)**			
		**All ages**	**11-18 y**	**19-64 y**	**65+ y**	**All ages**	**11-18 y**	**19-64 y**	**65+ y**
**2008-2009 (Year 1)**	1646	56 (299/533)	67 (79/118)	56 (148/266)	48 (38/80)	21 (110/533)	21 (25/118)	22 (58/266)	25 (20/80)
**2009-2010 (Year 2)**	1669	62 (394/631)	66 (93/141)	64 (214/335)	67 (58/87)	25 (155/631)	24 (34/141)	28 (93/335)	28 (24/87)
**2010-2011 (Year 3)**	1565	63 (352/561)	66 (85/129)	64 (192/300)	71 (45/63)	25 (138/561)	27 (35/129)	27 (82/300)	22 (14/63)
**2011-2012 (Year 4)**	1948	62 (460/737)	67 (91/135)	64 (269/420)	61 (66/108)	28 (209/737)	30 (41/135)	31 (132/420)	23 (25/108)
**2012-2013 (Year 5)**	1197	62 (291/469)	62 (44/71)	64 (167/260)	66 (56/85)	26 (121/469)	30 (21/71)	27 (69/260)	28 (24/85)
**2013-2014 (Year 6)**	1349	59 (300/509)	60 (63/105)	63 (162/256)	58 (49/86)	27 (138/509)	30 (31/105)	29 (74/256)	32 (27/85)
**2014-2015 (Year 7)**	1353	56 (259/462)	56 (49/88)	60 (148/246)	58 (47/81)	23 (107/462)	24 (21/88)	24 (60/246)	26 (21/81)
**2015-2016 (Year 8)**	1370	55 (278/504)	69 (60/87)	59 (161/274)	41 (28/69)	24 (120/504)	37 (32/87)	25 (68/274)	17 (12/69)
**2016-2017 (Year 9)**	1253	46 (194/425)	57 (39/69)	48 (110/228)	48 (32/67)	19 (82/425)	9 (20/69)	10 (47/228)	8 (14/67)

*Total number of participants surveyed (not all provided blood sample). EFSA, European Food Safety Authority; UK, United Kingdom; US, United States; y, years; 25(OH)D, 25-hydroxyvitamin D. Data in brackets represents number of participants classified as deficient/number of participants who provided blood sample for 25(OH)D analysis (standardised liquid chromatography coupled to tandem mass spectrometry (LC-MS/MS)).
